# Endovascular Intervention for a Carotid-Cavernous Fistula: A Case Report

**DOI:** 10.7759/cureus.44902

**Published:** 2023-09-08

**Authors:** Beshayer A Al-Boqami, Rahaf S Tammar, Sultan E Alharbi, Zahraa M Ahmed, Ahlam Alharbi

**Affiliations:** 1 General Practice, Al-Badaya General Hospital, Al Badayea, SAU; 2 Medicine, King Abdulaziz University, Jeddah, SAU; 3 General Practice, King Fahd Specialist Hospital, Buraydah, SAU; 4 Medicine, Zhejiang University, Hangzhou, CHN; 5 Family Medicine, Primary Health Care Center, Riyadh, SAU

**Keywords:** carotid-cavernous fistula, case report, digital subtraction angiography, computed tomography, superior ophthalmic vein, caroticocavernous fistula

## Abstract

A carotid-cavernous fistula is a rare vascular anomaly involving abnormal communication between the carotid artery and the cavernous sinus. This condition leads to the shunting of arterial blood directly into the venous system, causing diverse clinical manifestations. The classification includes direct and indirect fistulas, with endovascular techniques emerging as a preferred treatment option.

In this report, we present the case of a 58-year-old male who presented with progressive right-sided proptosis, headache, and visual disturbances. He exhibited right abducens nerve palsy, reduced visual acuity, and a dilated superior ophthalmic vein on imaging. A multidisciplinary team confirmed the diagnosis of a carotid-cavernous fistula and chose to pursue endovascular embolization. Catheter angiography revealed the fistula and balloon-assisted occlusion restored normal arterial flow. The patient's symptoms improved, and follow-up showed complete resolution of proptosis and enhanced visual acuity. Successful endovascular embolization underscores the significance of a multidisciplinary approach and showcases the positive outcomes achievable when diverse specialties converge for patient well-being.

## Introduction

A carotid-cavernous fistula is a rare vascular anomaly characterized by abnormal communication between the carotid artery and the cavernous sinus. This condition leads to the shunting of arterial blood directly into the venous system, resulting in a spectrum of clinical manifestations ranging from ophthalmic symptoms to potentially life-threatening complications [1]. Carotid-cavernous fistulas can be classified into various subtypes based on their etiology and hemodynamics, with direct and indirect carotid-cavernous fistulas being the most common types. Direct carotid-cavernous fistulas typically result from traumatic injuries, while indirect carotid-cavernous fistulas often arise spontaneously from the rupture of carotid artery aneurysms or cavernous venous malformations [1-2].

Endovascular techniques have become the preferred treatment modality for carotid-cavernous fistulas, leading to the successful closure of abnormal fistulous communication [2]. In this report, we present the case of a 58-year-old male with a carotid-cavernous fistula successfully managed by endovascular treatment. The case highlights the significance of endovascular interventions in effectively addressing carotid-cavernous fistulas and underscores the importance of multidisciplinary collaboration in achieving optimal patient outcomes.

## Case presentation

We present the case of a 58-year-old male who presented to our tertiary care medical center with a chief complaint of progressively worsening right-sided proptosis, headache, and visual disturbances over the past two months. He had a history of hypertension, which was well controlled with antihypertensive medications. There was no history of trauma or recent infections. Upon further inquiry, he reported a pulsatile tinnitus in the right ear, along with intermittent episodes of diplopia and blurring of vision. The patient denied any history of previous eye or neurologic issues.

On physical examination, the patient appeared alert and oriented. His vital signs were within normal limits. The neurological examination revealed a cranial nerve examination notable for right abducens nerve palsy and right-sided proptosis. Visual acuity was reduced in the right eye, with a visual acuity of 20/40 and 20/20 in the left eye. Fundoscopic examination revealed mild optic disc edema in the right eye. Extraocular movements were restricted in the right eye, particularly in the lateral gaze.

**Figure 1:**
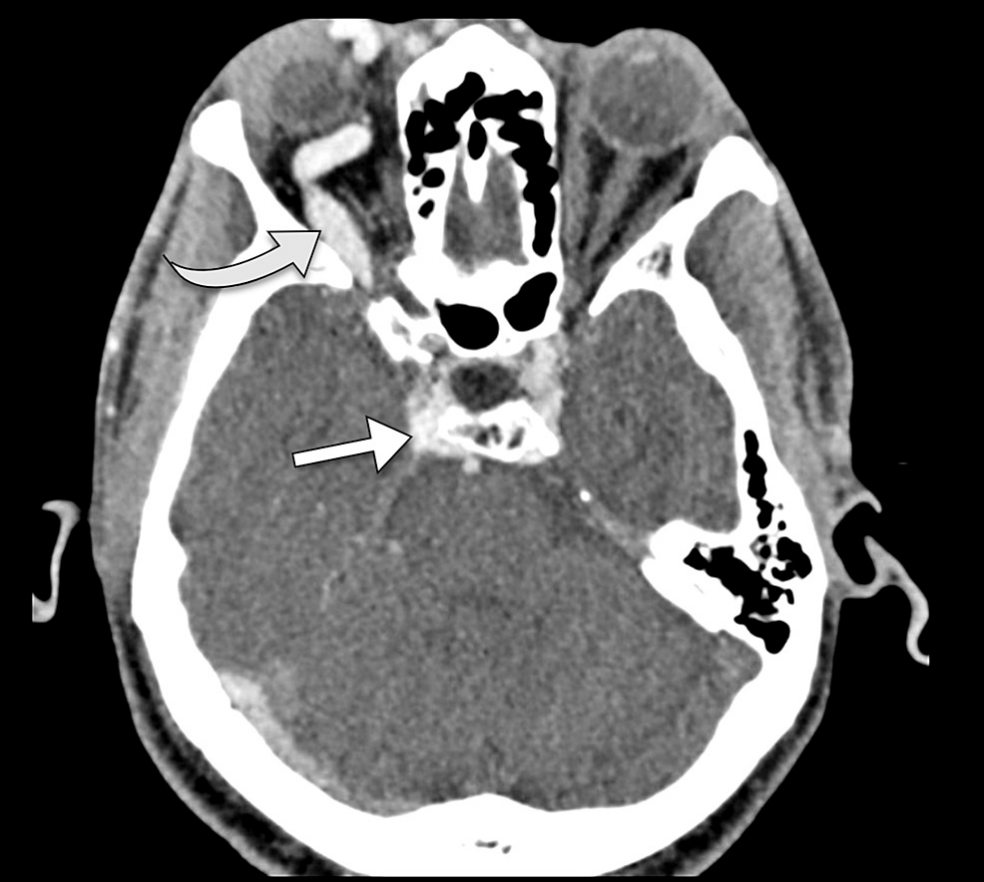
A contrast-enhanced axial CT image depicting a distended right cavernous sinus (arrow), accompanied by an enlarged right superior ophthalmic vein (curved arrow), is indicative of a right carotid-cavernous fistula.

Given the clinical presentation, a differential diagnosis was formulated, including conditions such as carotid-cavernous fistula, cavernous sinus thrombosis, orbital pseudotumor, and intracranial mass lesions. To further evaluate the patient, a comprehensive workup was initiated. Hematological investigations, including the complete blood count and coagulation profile, were within normal limits. A contrast-enhanced computed tomography scan of the head and orbits was performed, revealing dilatation of the superior ophthalmic vein and enlargement of the cavernous sinus on the right side (Figure 1).

The diagnosis of the carotid-cavernous fistula was thus confirmed based on the characteristic clinical presentation and the findings from the computed tomography scan. The management plan was discussed with the patient, and a multidisciplinary team consisting of neurosurgeons, interventional radiologists, and ophthalmologists was involved in his care. Given the presence of symptoms and potential complications, a decision was made to proceed with endovascular embolization.

**Figure 2:**
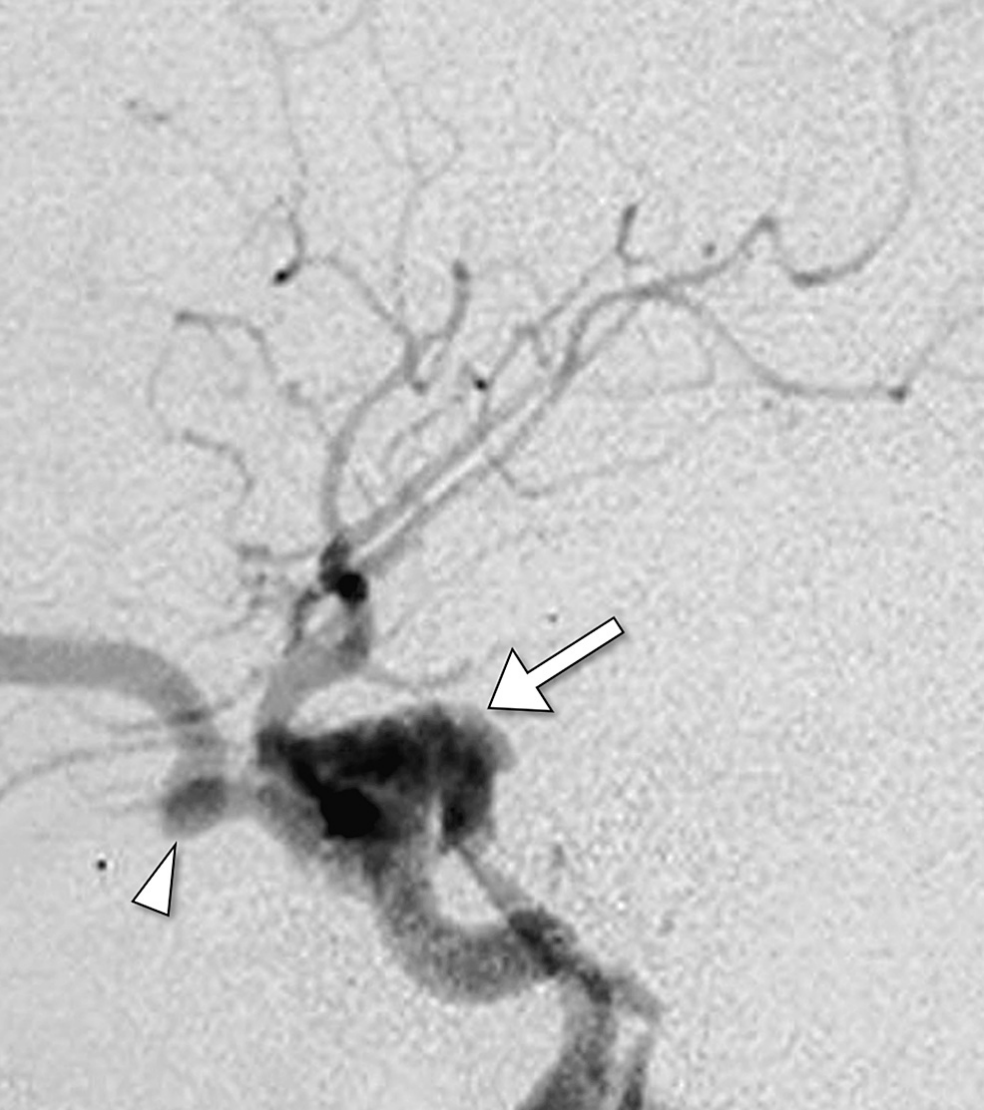
Catheter angiography involving injection of the right internal carotid artery, revealing contrast filling within the right cavernous sinus (arrow), along with retrograde flow extending to the dilated superior ophthalmic vein (arrowhead), confirming the presence of a right carotid-cavernous fistula.

**Figure 3:**
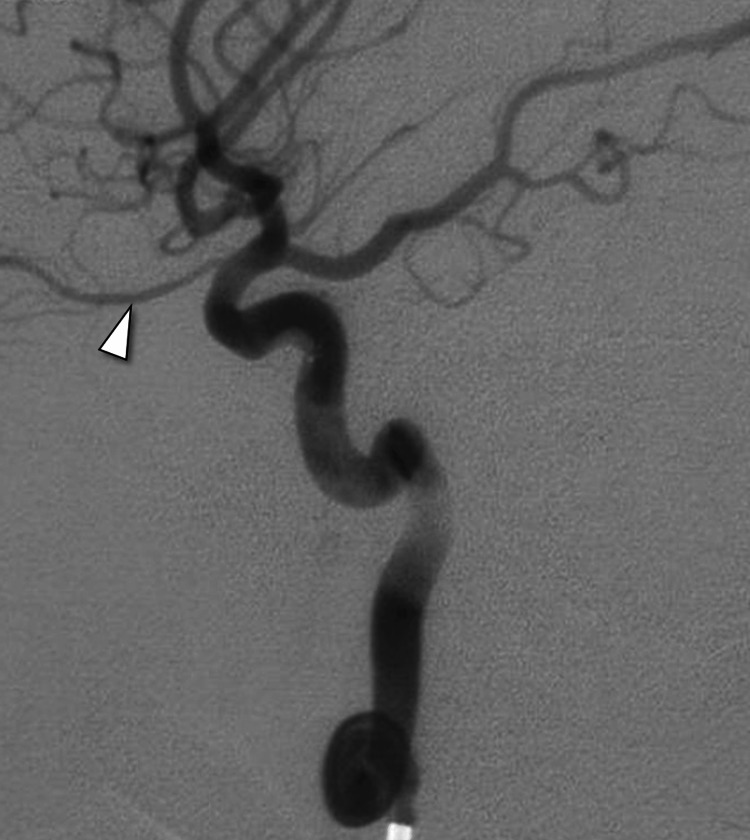
Subsequent catheter angiography following the balloon placement illustrating the restoration of a normal internal carotid artery contour, leading to enhanced flow into the ophthalmic artery (arrowhead).

Subsequently, digital subtraction angiography was performed to assess the vascular anatomy and confirm the diagnosis. Catheter angiography with injection of the right internal carotid artery revealed contrast filling the right cavernous sinus and draining across to the left side and down to the pterygoid venous plexus. Retrograde flow from the cavernous sinus distended the superior ophthalmic vein. Additional catheter angiography images obtained after placement of a balloon across the carotid artery defect showed restoration of the normal internal carotid artery contour. This intervention led to improved flow to the distal intracranial circulation, including the posterior communicating artery and the ophthalmic artery (Figures 2-3).

The patient's hospital course was uneventful, with a gradual resolution of right-sided proptosis and improvement in visual symptoms.

Follow-up visits were scheduled at regular intervals. At the three-month follow-up, the patient's right-sided proptosis had completely resolved, and his visual acuity had improved to 20/20 in the right eye. The abducens nerve palsy had also significantly improved. Clinical assessment and angiography confirmed the stable occlusion of the carotid-cavernous fistula, and the patient continued to show satisfactory recovery.

Following the successful endovascular embolization procedure, a structured post-operative treatment protocol was implemented. The patient had specific guidance on head positioning, and activity restrictions were provided to optimize postoperative recovery. The follow-up schedule included regular assessments to monitor ocular improvement, with evaluations performed at weekly intervals. Ophthalmic examinations were conducted during these follow-up visits, encompassing assessments of visual acuity, cranial nerve function, and any relevant ocular symptoms.

## Discussion

Carotid-cavernous fistulas are abnormal vascular connections between the carotid artery and the cavernous sinus. They are classified as either direct (high-flow) or indirect (low-flow) fistulas. Direct carotid-cavernous fistulas typically result from traumatic injuries, while indirect carotid-cavernous fistulas are often spontaneous and associated with conditions such as hypertension, atherosclerosis, and connective tissue disorders [1]. The presented case highlights a patient with an indirect carotid-cavernous fistula, as there was no history of trauma or recent infections, and the patient had a history of hypertension.

The patient's clinical presentation of right-sided proptosis, headache, visual disturbances, and cranial nerve deficits raised the suspicion for an underlying vascular abnormality. Differential diagnoses such as cavernous sinus thrombosis, orbital pseudotumor, and intracranial mass lesions were considered due to their overlapping symptomatology [3]. The importance of meticulous history-taking and a thorough physical examination, including cranial nerve assessment, is evident in reaching an accurate diagnosis.

In suspected carotid-cavernous fistula cases, imaging plays a pivotal role in confirming the diagnosis. Contrast-enhanced computed tomography scans can provide valuable information about vascular changes and sinus enlargement [3]. However, digital subtraction angiography remains the gold standard for diagnosing and characterizing carotid-cavernous fistulas, allowing visualization of the vascular anatomy and flow dynamics [4]. In the presented case, computed tomography findings were suggestive, but digital subtraction angiography was essential to confirm the diagnosis and guide further management decisions.

Carotid-cavernous fistula management requires a multidisciplinary approach involving neurosurgeons, interventional radiologists, and ophthalmologists. Treatment goals include relieving symptoms, preventing complications, and preserving visual function. The decision to proceed with endovascular embolization in this case highlights the importance of carefully weighing the risks and benefits of available interventions [2].

The management of carotid-cavernous fistulas involves distinct approaches for direct and indirect types. Direct carotid-cavernous fistulas, characterized by high flow and prominent symptoms, are treated with endovascular techniques aimed at blocking abnormal communication while preserving the carotid artery [5-6]. This typically involves the use of detachable coils, liquid embolic agents, and sometimes arterial stents. In rare cases, the internal carotid artery may need to be occluded [6]. Indirect carotid-cavernous fistulas, which have low-flow and milder symptoms, are only treated when symptoms are severe or vision is threatened. The preferred approach for indirect carotid-cavernous fistulas is transvenous embolization, often through the inferior petrosal sinus, using coils, liquid embolic agents, or a combination of both. Ethylene vinyl alcohol copolymer (Onyx®) is a potential superior agent in some cases [6].

The resolution of the patient's symptoms and improvement in clinical parameters following endovascular embolization underscore the effectiveness of the chosen intervention. Successful treatment not only addresses the patient's immediate concerns, such as proptosis and visual disturbances, but also prevents potential complications, such as intracranial hemorrhage, cranial nerve palsies, and severe visual loss. The multidisciplinary team's coordination and expertise played a crucial role in achieving these positive outcomes [2,5].

The case's three-month follow-up demonstrates the importance of long-term monitoring for potential recurrence or complications. Stable occlusion of the fistula and sustained improvement in symptoms indicate a favorable prognosis. However, continued follow-up is crucial to detect any late complications or residual effects, ensuring the patient's continued well-being [5].

## Conclusions

In conclusion, this case underscores the significance of meticulous clinical assessment and interdisciplinary collaboration in diagnosing and managing carotid-cavernous fistulas. The successful resolution of the patient's symptoms through endovascular embolization highlights the advancements in modern medical techniques and the pivotal role of a multidisciplinary approach. This case serves as a reminder to healthcare professionals of the importance of considering vascular anomalies in patients with atypical symptoms and reinforces the positive outcomes achievable when expertise from diverse specialties converges for the well-being of the patient.
